# Iron Overload Is Associated With Accelerated Progression of Osteoarthritis: The Role of DMT1 Mediated Iron Homeostasis

**DOI:** 10.3389/fcell.2020.594509

**Published:** 2021-01-05

**Authors:** Xingzhi Jing, Jiamin Lin, Ting Du, Zhensong Jiang, Tao Li, Guodong Wang, Xiaoyang Liu, Xingang Cui, Kai Sun

**Affiliations:** ^1^Department of Spine Surgery, Shandong Provincial Hospital Affiliated to Shandong First Medical University, Jinan, China; ^2^Department of Orthopedics, Tongji Hospital, Tongji Medical College, Huazhong University of Science and Technology, Wuhan, China; ^3^Department of Otolaryngology–Head and Neck Surgery, Shandong Provincial ENT Hospital Affiliated to Shandong University, Jinan, China; ^4^Department of Spine Surgery, Shandong Provincial Hospital, Cheeloo College of Medicine, Shandong University, Jinan, China

**Keywords:** iron overload, osteoarthritis, divalent metal transporter 1 (DMT1), inflammation, chondrocyte

## Abstract

**Objective:** Iron overload is common in elderly people which is associated with an increased prevalence of osteoarthritis (OA), but the exact role of iron in the development of OA has not been established. The aim of the present study is to elucidate the connection between iron overload and OA using an iron overloaded mice model, as well as to explore the role of iron homeostasis, iron transporters dependent iron influx in OA pathogenesis.

**Methods:** The iron overloaded mice model was established and OA was surgically induced. OA progression was assessed at 8 weeks after surgery. Next, primary chondrocytes were treated with pro-inflammatory cytokines and iron regulators mediated iron homeostasis were evaluated. Involvement of iron transporters was analyzed using chondrocytes mimicking an osteoarthritis-related phenotype *in vitro*.

**Results:** Iron overloaded mice exhibited greater cartilage destruction and elevated ADAMTS5 as well as MMP13 expression along with increased iron accumulation and dysregulated iron regulators. Pro-inflammatory cytokines could disturb cellular iron homeostasis via upregulating iron import proteins, TFR1 and DMT1, downregulating iron efflux protein FPN, thus result in cellular iron overload. Among iron transporters, DMT1 was found to play pivotal roles in iron overload induced OA progress. Inhibition of DMT1 suppressed IL-1β induced inflammatory response and ECM degradation via blockade of MAPK and PI3K/AKT/NF-κB pathways.

**Conclusions:** Our results suggest that iron takes parts in the development of OA and cutting iron influx via inhibiting DMT1 activity could be an attractive new target for OA treatment.

## Introduction

Osteoarthritis (OA) is a chronic degenerative joint disease which is characterized by progressive loss of joint cartilage and subchondral bone remodeling. Although age, mechanical overload, joint injuries and gender are major risk factors for the disease, the intrinsic physiological and molecular mechanisms of OA remain poorly understood (Glyn-Jones et al., 2015). Hereditary hemochromatosis (HH) is caused by mutations in the HFE gene and characterized by systemic iron overload in many tissues, including liver, heart, kidney, brain, and bone. Patients suffering from HH frequently develop osteoarthritis, raising a growing interest in the potential role of iron in the pathogenesis of OA (van Vulpen et al., 2015a).

Iron is essential metal ions in the human body and plays important physiological and biological roles in oxygen transport, DNA synthesize, energy metabolism, and hundreds of enzymes synthesize (Gozzelino and Arosio, 2016; Tang et al., 2018). However, excess free iron could act as a catalyst to produce large amount of reactive oxygen species (ROS) and lead to oxidant mediated cellular injury (Xie et al., 2020). Because its double-edged sword effect in the body, iron homeostasis is delicately regulated. Cellular iron homeostasis is achieved by modulating the expression of proteins involved in iron uptake, storage, and export (Kuhn, 2015). Iron metabolism regulating proteins (IRPs) sense the concentration of active iron in the mitochondria and regulate the expression of iron uptake associated proteins transferrin receptor 1(TFR1) and divalent metal transporter 1 (DMT1) (Zhang et al., 2014). IRP could also bind to the IRE of the 5' untranslated region of ferroportin (FPN) and regulate the iron release from the cell (Wang et al., 2019). Disruption of iron homeostasis is reported to take parts in many diseases, such as Parkinson disease, Alzheimer disease, cardiovascular diseases and osteoporosis (Siddique and Kowdley, 2012).

Because the human body lacks effective ways to excrete excess iron, many risk factors including aging, genetic mutations, dietary iron intake, and chronic blood transfusion will lead to progressive and pathological iron overload of the body (Chen et al., 1998; Jeney, 2017). Although iron is essential for human life, increasing evidence shows a link between iron overload and OA (Nugzar et al., 2018). Diseases/conditions with diverse etiology, such as hemoglobinopathies, hereditary hemochromatosis and aging, could lead to chronic iron accumulation (Siddique and Kowdley, 2012; Krishan et al., 2015). Besides genetic and metabolic dysfunction, clinical studies indicate excess iron accumulation in joints of age-related osteoarthritis, rheumatoid arthritis and traumatic arthritis. In a 2-year longitudinal observational study, Kennish L et al. found that serum iron levels were positively correlated with OA, moreover, analysis of radiographic data indicated that higher ferritin is associated with higher risk prediction of radiographic severity (Kennish et al., 2014). Hereditary hemochromatosis (HH) is a chronic systemic iron overloaded disease which is caused by mutations in the *Hfe* gene. *Camacho A et al*. demonstrated that HH was associated with an accelerated development of OA and synovial hemosiderin deposits originated from microbleeding or oozing from inflamed synovial tissue had a definite role in the progression of HH-related OA (Camacho et al., 2016).

The association of iron overload with OA pathogenesis is broadly appreciated, however, it is still unclear whether iron takes parts in OA pathogenesis. Moreover, how iron homeostasis is regulated during the onset and progression of OA and how it contributes to the pathological transition of articular chondrocytes remain unknown. In the present study, we first established iron overloaded mice model to investigate whether iron overload contributes to the onset and progression of OA. Next, the role of iron homeostasis, iron regulators in OA pathogenesis was investigated. We found that disruption of iron homeostasis took parts in the onset and progression of OA and DMT1 was found to play pivotal roles in iron overload induced OA progression.

## Materials and Methods

### Reagents

Recombinant mouse IL-1β or TNF-α were purchased from R&D systems (Minneapolis, MN, USA). Ferric ammonium citrate (FAC), Deferoxamine (DFO) were purchased from Sigma Aldrich. Rabbit antibodies specific for transferrin receptor (TfR1, ab84036), DMT1 (ab55735), Ferroportin (FPN, ab78066), MMP13 (ab39012), ADAMTS5 (ab182795), SOX9 (ab185966), COL2 (ab185570) were purchased from Abcam (Cambridge, UK). Rabbit polyclonal antibodies specific for MMP3 (17873-1-AP), GAPDH (60004-1-Ig) were purchased from Proteintech Group (Wuhan, China). Antibodies against iNOS (#sc-7271), ERK1/2 (#4695), Phospho-ERK (#4370), JNK (#9258), Phospho-JNK (#9255), P38(#8690), Phospho-P38 (#9216), p65 (#8242), Phospho-p65 (#3033) were supplied by Cell Signaling Technology Inc. (Beverly, MA, USA).

### Animal Grouping and Treatment

Ten two-month-old C57/BL6 male mice were purchased from the Experimental Animal Center of Tongji Medical College (Wuhan, China). The animals were randomly divided into two groups: control group and iron overloaded group. Only male mice were used in this study to avoid estrogen interference. The iron overloaded mice model was established according to previous studies (Tsay et al., 2010). Briefly, Mice in iron overload group were treated intraperitoneally once a week for 2 months with iron dextran (500 mg/kg). Two weeks after the first injection of iron dextran, OA was induced in the right knee joints of control group and iron overloaded group by transection of the anterior attachment of the medial meniscus to the tibial plateau (DMM) using a microscope. The left knees were sham-operated (SHAM). All experiments were approved by the Institutional Animal Care and Use Committee (IACUC) of Tongji Hospital.

### Assessment of Iron Content

To assess iron content in the liver and blood, five animals of each group were used. After 2 months of DMM surgery, the mice were anesthetized, blood was collected by intra-cardiac puncture for determination of serum iron, liver tissue was collected to determine the hepatic iron concentration (Rebouche et al., 2004). Briefly, for serum iron, the specimen was centrifuged at 1,500–2,000 g for 45 min and the serum was decanted carefully to minimize contamination with erythrocytes. Thirty microlitre serum was treated with 30 μL protein precipitation solution (1 mol/L hydrochloric acid and 10% trichloroacetic acid). For liver iron, 10 mg dry liver tissue homogenates were prepared in 100 ml high-purity water, then 30 μL tissue homogenates were treated with equal volume of protein precipitation solution and vortex mixed. Then the mixture was heated in 95°C for 1 h to release iron ions. After centrifuged at 8,200 g for 10 min, 30 μL supernatant were treated with 30 μl of chromogen solution (0.508 mmol/L ferrozine, 1.5 mol/l sodium acetate and 0.1% or 1.5% (v/v) thioglycolic acid in high purity water). After 30 min at room temperature, absorbance was measured at 562 nm using spectral IMAX190 absorbance microplate reader (Molecular Device, USA) and iron content was calculated according to standard curves which were prepared using iron standards containing 0, 2, 4, 6, 8 and 10 μg/ml of the iron standard solution.

### Micro-Computed Tomography (CT) Analysis

After 2 months of DMM surgery, all mice were euthanized and bilateral knee joints from 30 mice were isolated and fixed in 4% paraformaldehyde for 24 h. The microarchitecture of joints was scanned with micro-computed tomography (μCT, viva CT 40, Scanco 274 Medical, Switzerland) at a resolution of 10.5 μm, 100 kV, 98 μA. Three-dimensional reconstruction and data processing were accomplished using the built-in software. We chose the medial tibial subchondral bone for 3D reconstruction and structural parameters analysis. Direct 3D measurement methods were used to calculate the following parameters: bone volume per tissue volume (BV/TV), trabecular separation (Tb.sp), trabecular thickness (Tb.Th) and Connective density (Conn.D, in mm^−3^), a measurement of the average number of trabeculae per unit of volume.

### Histological Staining and Analysis

Following micro-CT the samples were decalcified with 10% EDTA solution at 4°C for 2 weeks, and then embedded in paraffin wax. Five micrometer sagittal sections were cut from the medial compartment of the joints and stained with Safranin-O/Fast Green. Osteoarthritis Research Society International (OARSI) served as histopathology scoring system to evaluate severity of OA changes. Tartrate-resistant acid phosphatase (TRAP) staining (Sigma-Aldrich, CA) was performed to analysis the osteoclasts formation and the number of osteoclasts/bone surface (N.Oc/BS) was measured as previously reported (Jing et al., 2019). To assess iron deposition in joints, Perl's Prussian blue staining was used to detect hemosiderin deposits using commercial Perl's Prussian blue staining kit (G1426, life sciences). Briefly, Perl's stain was prepared by mixing Perl's stain A1 and A2, 5 μm sagittal sections were cut from the medial knee compartment of the decalcified samples and deparaffinized, then incubated with Perl's stain at 37°C for 30 min, after washed by water twice for 2 min, Perl's stain B was added and incubated at room temperature for 5 min, then washed by water twice. The number of iron deposits was counted in the cartilage and synovial tissue area per specimen.

### Immunohistochemistry

From these decalcified samples, 5 μm sagittal sections were cut from the medial knee compartment and were deparaffinized, antigen retrieved, incubated with corresponding primary antibodies TFR1 (ab84036, Abcam, dilution 1:3000), DMT1 (ab55735, Abcam, dilution 1:1000), FPN(ab78066, Abcam, dilution 1:100), MMP13 (ab39012, Abcam, dilution 1:1000), ADAMTS5 (ab182795, Abcam, dilution 1:1000), and then incubated with biotinylated goat anti-rabbit (BA1003, Boster, China, dilution 1:100) secondary antibodies. Sections were colored with DAB and counterstained with hematoxylin. The images of immunohistochemical staining were analyzed using the software Image-Pro Plus. To quantify immuno-positive cells, at least five random fields in the cartilage were selected and the ratio of immune positive cells to total cells were calculated.

### Cell Isolation and Culture

Cartilage was acquired from the knee joints of 5 days old C57/BL6 male mice and minced into small pieces. After digested with 0.25% trypsin and 0.25% collagenase solution. Cells were collected and cultured in DMEM/F12 (Hyclone, Life sciences, USA) medium containing 10% FBS (Gibco; Thermo Fisher Scientific, Inc., USA) in a humidified atmosphere of 5% CO_2_ at 37°C. Chondrocytes were triangle and arranged closely under microscope. Cells were passaged when up to 80% and chondrocytes at passage one and two were used in this study.

### Calcein Loading of Cells and Iron Influx and Efflux Assay

Iron influx into chondrocytes was determined by the quenching of calcein fluorescence as described before (Tenopoulou et al., 2007). The Calcein-AM (calcein-acetoxymethyl ester) method is a widely used technique that is supposed to assay the intracellular “labile iron pool” (LIP). Calcein-AM could easily penetrate cell membrane, the esterase will hydrolyze it into Calcein and emit strong green fluorescence. Calcein fluorescence could be quenched following chelation of labile iron and the degree of quenching could reflect the amount of intracellular labile iron. Briefly, Primary chondrocytes were seeded in 24-well plate at a concentration of 1 × 10^5^cells/ml. Twenty-four hours later, serum-free medium with 10 ng/mL IL-1β or TNF-α was changed and cultured for 24 h, then cells were loaded with 0.5 μmol/L Calcein-AM for 30 min at 37°C. Calcein fluorescence was recorded at 488 nm excitation and 525 nm emission wavelengths, and fluorescence intensity was measured every 5 min for 5 times while perfusing with 0.1 mmol/L ammonium ferric citrate (FAC). For iron efflux assay, chondrocytes were perfused with 0.1 mmol/L FAC for 20 min, then 1 mmol/L DFO was added to chelate iron ions and the intracellular iron was drained out to the medium, indicated by the increase in calcein fluorescence(Wang et al., 2013). Fluorescence intensity was measured at 488 nm excitation and 525 nm emission wavelengths every 5 min for 5 times with fluorescence microscope (Evos fl auto; Life Technologies, Carlsbad, CA). The mean fluorescence signal of 25–30 single cells in five separate fields was monitored at ×200 magnification and processed with Image Pro Plus.

### Western Blot Analysis

Briefly, cells were harvested and lysed with RIPA lysis buffer (Boster, China) supplemented with protease and phosphatase inhibitor. Cell homogenates were collected and centrifugated at 12,000 rpm for 20 min at 4°C. Next the total protein concentration was determined with BCA method. Twenty-five μg proteins were subjected to SDS-PAGE and transferred to 0.45-μm PVDF membranes (Millipore, Billerica, MA). Following blockage with 5% BSA for 1 h, membranes were incubated with primary antibodies anti TFR1 (ab84036, Abcam, dilution 1:1000), DMT1 (ab55735, Abcam, dilution 1:1000), FPN(ab78066, Abcam, dilution 1:1000), MMP13 (ab39012, Abcam, dilution 1:1000), ADAMTS5 (ab182795, Abcam, dilution 1:1000), MMP3 (17873-1-AP, Proteintech, dilution 1:1000), SOX9 (ab185966, Abcam, dilution 1:1000), COL2 (ab185570, Abcam, dilution 1:2000), iNOS (#sc-7271, CST, dilution 1:1000), ERK1/2 (#4695, CST, dilution 1:1000), Phospho-ERK (#4370, CST, dilution 1:1000), JNK (#9258, CST, dilution 1:1000), Phospho-JNK (#9255, CST, dilution 1:1000), P38(#8690, CST, dilution 1:1000), Phospho-P38 (#9216, CST, dilution 1:1000), p65 (#8242, CST, dilution 1:1000), Phospho-p65 (#3033, CST, dilution 1:1000),GAPDH (60004-1-Ig, Proteintech, dilution 1:10000) at 4°C overnight. The membranes were then washed with TBST and incubated with a horseradish peroxidase-conjugated secondary antibody (BA1055, Boster, China, dilution 1:10000) at room temperature for 1 h. The protein bands were visualized using Western ECL Substrate Kit (Thermo Pierce, USA) and analyzed with the built-in software of Bio-Rad scanner (Bio-Rad, Hercules, CA). Images were acquired with Bio-Rad scanner (Hercules, CA) and densitometry was quantified by digital image analysis software (Quantity One, Bio-Rad, Hercules, CA). GAPDH served as an internal control to normalize the results.

### Evaluation of Apoptosis by Annexin V-FITC/PI Staining

Annexin V-FITC/PI kit (BD Biosciences, Franklin Lakes, NJ) was used to investigate the apoptotic effect of FAC according to manufacturer's instructions. Chondrocytes were seeded into a 6-well plate at a concentration of 2 × 10^5^cells/ml, when reached 90% confluence, cells were treated with various concentrations of FAC (0–100 μM) for 24 h. After treatment, cells were harvested and washed with PBS twice. Then the cells were stained with Annexin V-FITC/PI at room temperature for 15 min in the darkness and then analyzed using a FACSCalibur flow cytometer (FACSort; BD Biosciences, Franklin Lakes, NJ). Annexin V^+^/PI^−^ and annexin V^+^/PI^+^ were considered as apoptotic cells in the early and late phase, respectively.

### *Dmt1* siRNA Transfection

Chondrocytes at the second passage were seeded at the density of 1 × 10^5^cells/ml in 6-well plates and cultured until cells reached 30% confluence. Following removal of the cell culture medium, cells were incubated with DMEM/F12 containing siDMT1 and siControl. SiRNAs targeting *Dmt1* mRNA and scrambled siRNA were synthesized and purchased from Ribo Bio (Ribobio Co. Ltd., Guangzhou., China). The efficiency of *Dmt1* silence was analyzed by RT-PCR analysis. 100 nM siRNA were transitorily transfected into chondrocytes by lipofectamine 3,000 (Invitrogen, Carlsbad, CA) and medium was replaced 24 h after transfection. Scrambled siRNA was used as negative control (NC). Following transfection, the medium was changed with DMEM/F12 and 10% FBS with or without 10 ng/ml IL-1β for 24 h, the total protein was extracted and western blot was conducted to assess the targeted proteins.

### Reverse Transcription and Real-Time Polymerase Chain Reaction (RT-PCR) Analysis

RT-PCR was used to quantify gene expression. Total RNA (mRNA) was extracted from articular chondrocytes using the total RNA extraction kit (Toyobo, Japan) following the manufacturer's instructions. RNA concentration and purity were assessed using a Microvolume Spectrophotometer (Thermo Fisher Scientific, Logan, UT, USA). Complementary DNA (cDNA) was synthesized from 1 μg of total RNA using first Strand cDNA Synthesis Kit (Toyobo, Osaka, Japan). Then the cDNA was amplified by SYBR Green Real-time PCR Master Mix (Toyobo, Osaka, Japan) with following cycling conditions: 30 s of polymerase activation at 95°C, followed by 40 cycles of 95°C for 5 s and 60°C for 30 s. GAPDH was used as an internal control. Each cDNA sample was run in triplicate. Sequences of primers for the reference gene (GAPDH) and interested genes are listed as follows: DMT1, Forward: 5′-TCTTCGGTTCCTCTCACTCCTGTG-3′, Reverse:5′-AGAGCCTGCCACCACCAGTC-3′; TFR1, Forward:5′-AATGGTTCGTACAGCAGCGGAAG-3′, Reverse:5′-TAGCACGGAAGTAGTCTCCACGAG-3′; FPN, Forward:5′-GCCTCTGCCTCTGCCTCTACC-3′, Reverse: 5′-AGTCAGTCCACGAGTGCTACAGTC-3′; GAPDH:Forward:5′-CTCCCACTCTTCCACCTTCG-3′, Reverse:5′-TTGCTGTAGCCGTATTCATT-3′

### Statistical Analysis

The comparisons between multiple groups, such as OARSI scores and immunohistochemistry analyses were performed using multiple comparisons by one-way ANOVA followed by Tukey's test. For western blot data expressed as relative fold changes, Student's *t*-test and one-way ANOVA with Dunnett's test were used for pairwise comparisons and multi-group comparison, respectively. Results are represented as mean ± s.d. *p* < 0.05 were considered to be significant. All analyses were performed with GraphPad Prism software (Version 6.0).

## Results

### Iron Overloaded Mice Had Increased Iron Accumulation in the Serum, Liver and Knee Synovial Membrane

**Figure 1:**
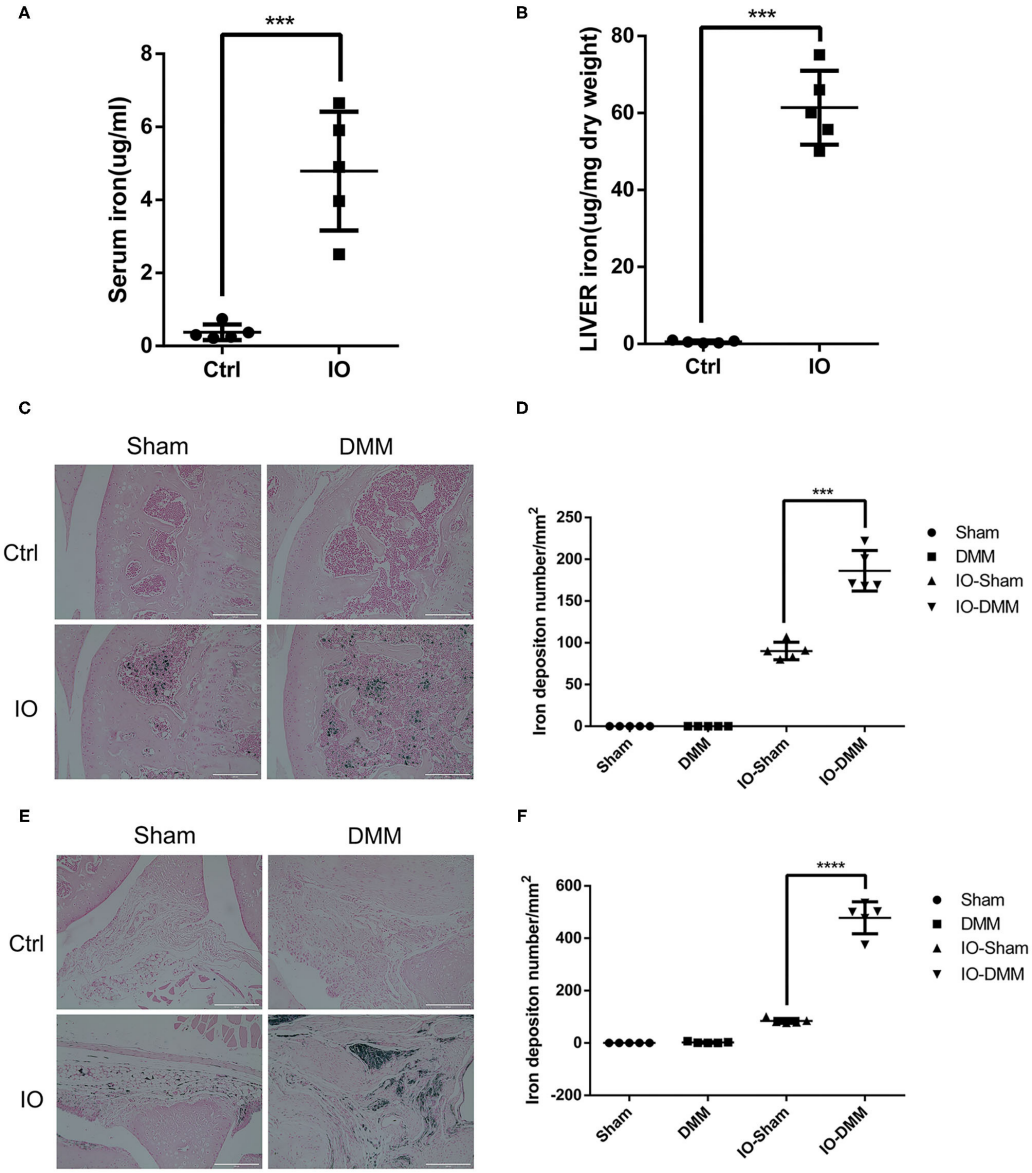
Iron parameters in control (Ctrl) and iron overloaded (IO) group (*n* = 5 per group). **(A,B)** Serum and liver iron content in control (Ctrl) and iron overloaded (IO) group. Data are presented as mean ±SD. ***P < 0.001. Effect size (EZ) = 0.885 for **(A)**, EZ=0.976 for **(B)**. Perl's Prussian blue staining for cartilage **(C)** and synovial tissue **(E)** of Sham and DMM operated knees of Ctrl group and iron overloaded (IO) group. Scale bar=200 μm. **(D,F)** The iron deposition number/mm^2^ was quantified and data are presented as mean ±SD. ***p < 0.001, ****p < 0.0001. EZ=0.932 for **(D)**, EZ=0.976 for **(F)**.

Iron dextran (0.5 g/kg/wk) was administrated intraperitoneally for 2 months to establish iron overloaded (IO) mice model. Serum and liver iron content were measured to examine the establishment of iron overloaded model. As expected, serum and liver iron content were markedly increased in iron overloaded mice with up to 12- and 110-fold when compared to their controls (Figures 1A,B). Next, Perl's prussian blue staining was performed to detect the hemosiderin deposition in the joints. To evaluate the effect of iron overload in the development of OA, we surgically induced OA in the right knees of iron overloaded mice and control mice using DMM surgery, the left knees were regarded as sham control. We observed large amounts of hemosiderin deposits in the subchondral bone marrow of iron overloaded (IO) mice, but Perl's prussian blue staining results showed no significant blue hemosiderin deposits in cartilage (Figures 1C,D). Perl's prussian blue staining of synovial tissue showed a large amount of hemosiderin deposits in synovial tissue in iron overloaded mice and the number of hemosiderin deposits in DMM operated right knees is five-fold of that in sham operated left knees (Figures 1E,F). These results indicate that hemosiderin deposits might originate from the blood that enters the joint. Greater iron accumulation might result from higher serum iron content of the iron overloaded mice.

### Iron Overloaded Mice Had Increased Cartilage Degeneration Following the Surgical Induction of OA

**Figure 2:**
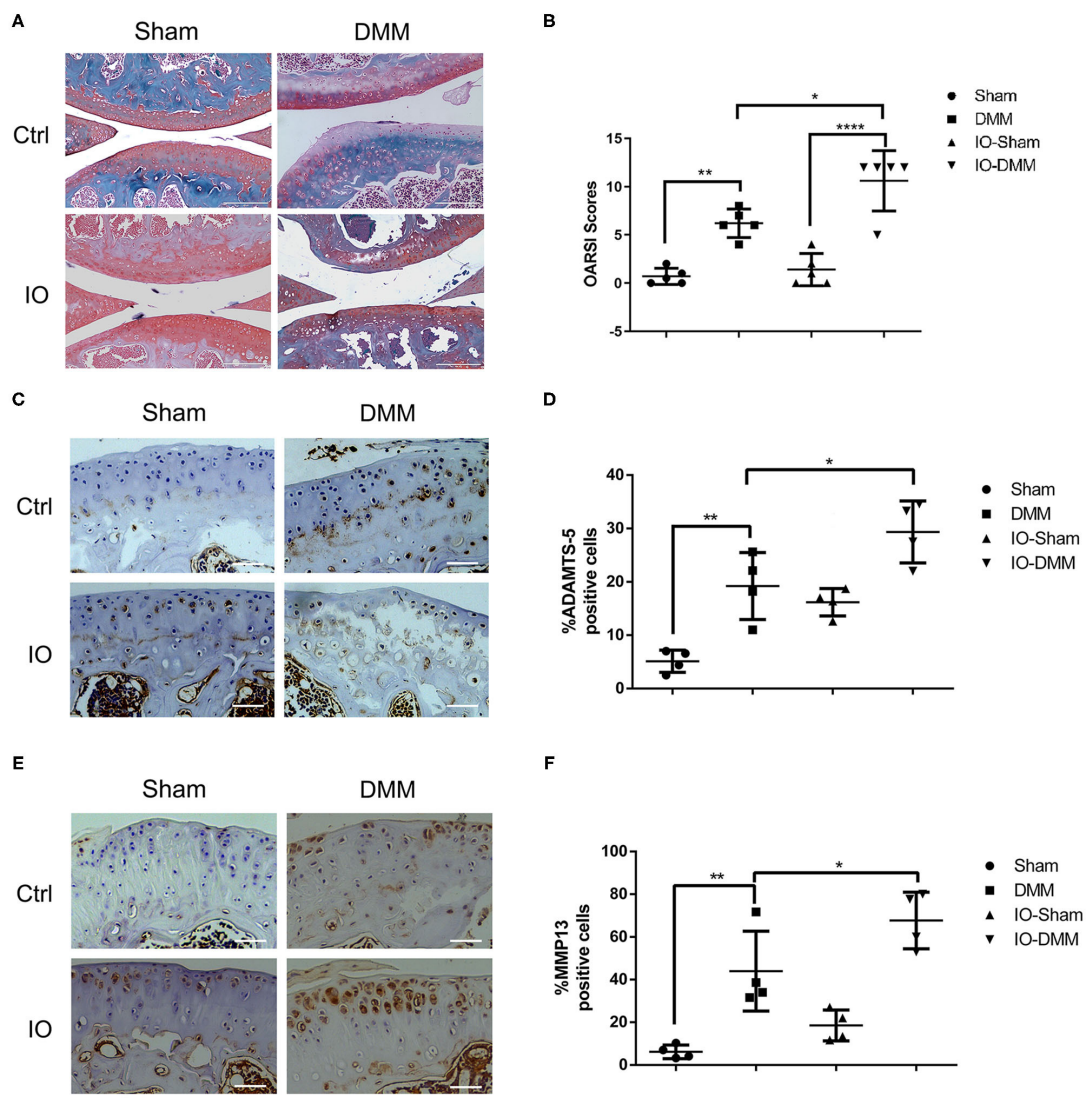
Cartilage changes and immunohistochemistry for matrix degrading enzymes ADAMTS5 and matrix metallopeptidase 13 (MMP-13) in Ctrl group and iron overloaded (IO) group. **(A)** Haematoxylin-Fast Green-Safranin-O staining of the medial femoral condyle and tibial plateau of control (*n* = 5) and iron overloaded (*n* = 5) mice at 2 months after surgical induction of OA. Iron overloaded mice showed a higher level of cartilage degeneration and higher OARSI scores in the right knees compared to the control group, while sham operated left knees of both two groups showed no significant cartilage destruction. **(B)** The Osteoarthritis Research Society International (OARSI) scores for the Sham and DMM operated knees of Ctrl group and iron overloaded (IO) group. Scale bar=200 μM; Data are presented as mean ± SD. *P < 0.05; **P < 0.01; ****P < 0.0001. EZ=0.838 for **(B)**. Immunohistochemistry for matrix degrading enzymes ADAMTS5 **(C)** and matrix metallopeptidase 13 (MMP-13) **(E)** in the medial tibial plateau at 2 months after surgical induction of OA in Ctrl group (*n* = 4) and iron overloaded group (*n* = 4). The ratio of positive cells for ADAMTS5 **(D)** and MMP13 **(F)** were quantified under a microscope at 40X magnification using five sections from four mice. Scale bar=50 μM; Data are presented as mean ± SD. *P < 0.05; **P < 0.01. EZ=0.826 for **(D)**; EZ=0.836 for **(F)**.

To evaluate the effect of iron overload in the development of OA, we surgically induced OA in control group and iron overloaded group by transection of the anterior attachment of the medial meniscus to the tibial plateau (DMM). After 8 weeks, the DMM-operated knees of both control group and iron overloaded group showed a significant cartilage degeneration and a higher summed tibial and femoral OARSI scores compared to the left knees, indicating that OA model was successfully established. We next assessed the effect of iron overload in the accelerated progression of OA. We found that iron overloaded mice showed a higher level of cartilage degeneration, resulting in higher OARSI scores in the right knees compared to the control group. Interestingly, sham operated left knees of both two groups showed no significant cartilage destruction, indicating iron overload alone seems not to be enough to cause OA in this time period (Figures 2A,B). We next detected matrix-degrading enzymes, such as ADAMTS5 and MMP13 using immunohistochemical staining. As shown in Figures 2C–F, both the DMM operated knees of iron overloaded and the control group had a higher percentage of articular chondrocytes positive for ADAMTS5 and MMP13 compared to sham operated knees, and DMM operated knees of iron overloaded mice displayed stronger staining and greater percentage of ADAMTS5 and MMP13 positive cells compared to that of control group (Figures 2C–F).

### Iron Overloaded Mice had Increased Amount of Osteoclasts and Subchondral Bone Volume

**Figure 3:**
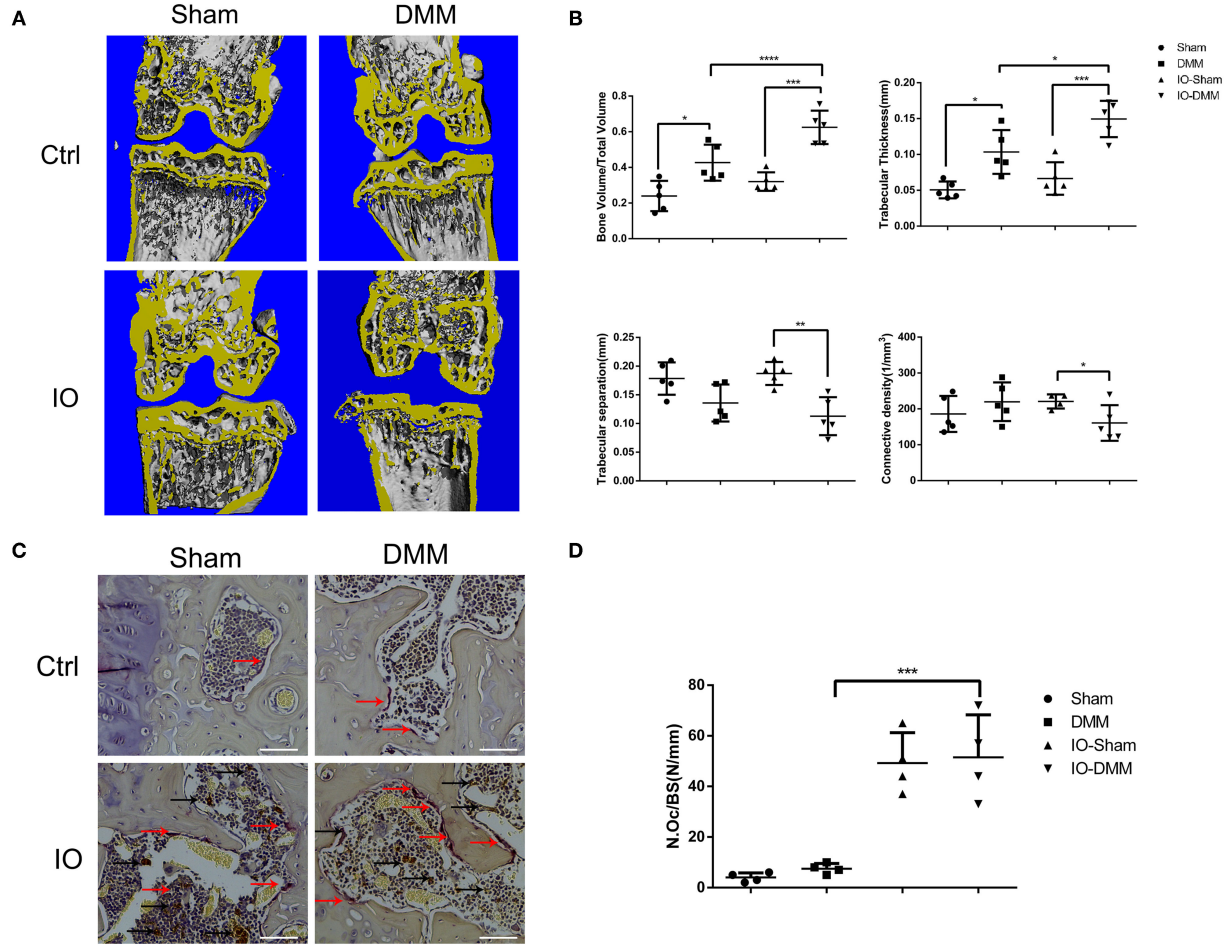
Iron overload promoted subchondral bone sclerosis and osteoclasts formation. **(A)** Micro-CT analysis of subchondral bone changes following DMM surgery in Ctrl group and iron overloaded (IO) group. **(B)** Quantification of microarchitecture parameters [bone volume per tissue volume (BV/TV), trabecular separation (Tb.sp), trabecular thickness (Tb.Th) and Connective density (Conn.D, in mm^−3^)] of the medial tibial plateau of mice knees (*n* = 5 per group). The results showed that the DMM operated knees of the iron overloaded mice had a greater degree of subchondral sclerosis when compared to that of Ctrl group. **(C)** Representative TRAP-stained histological sections of subchondral bone 2 months after surgical induction of OA in Ctrl group and iron overloaded group (*n* = 4 per group). Red arrows indicated osteoclasts. Black arrows indicated iron deposits. **(C)** Scale bars=100 μM. Data are presented as mean ± SD. *P < 0.05; **P < 0.01; ***P < 0.001; ****P < 0.0001. EZ=0.783 for BV/TV; EZ=0.766 for Tb.sp; EZ=0.582= Tb.Th; EZ=0.256 for Conn.D. EZ=0.86 for **(D)**.

Subchondral bone architecture plays important roles in the pathogenesis progression of OA (Aho et al., 2017; Finnila et al., 2017). In the present study, to better evaluate the subchondral bone architecture of the medial tibial plateau, micro-CT scans of the joints were performed. The DMM operated right knees of iron overloaded mice showed obvious subchondral bone sclerosis with higher BV/TV, trabecular thickness and lower Tb.Sp, Conn.D (Figures 3A,B). Iron overload was reported to be able to promote osteoclasts formation and was responsible for bone loss of osteoporosis in old people. To evaluate the effect of iron overload in subchondral osteoclast formation. The TRAP staining assay was performed to examine the osteoclast formation. As shown in Figures 3C,D, the amounts of TRAP positive osteoclasts were significantly increased in iron overloaded mice group with about 10-fold compared to control group, which could explain the subchondral bone collapse and the subsequent bone sclerosis.

### The Pro-inflammatory Cytokines Upregulated Iron Influx Mediators TFR1 and DMT1, Downregulated Iron Efflux Mediator FPN in Chondrocytes Under Pathological Conditions and in OA Cartilage

**Figure 4:**
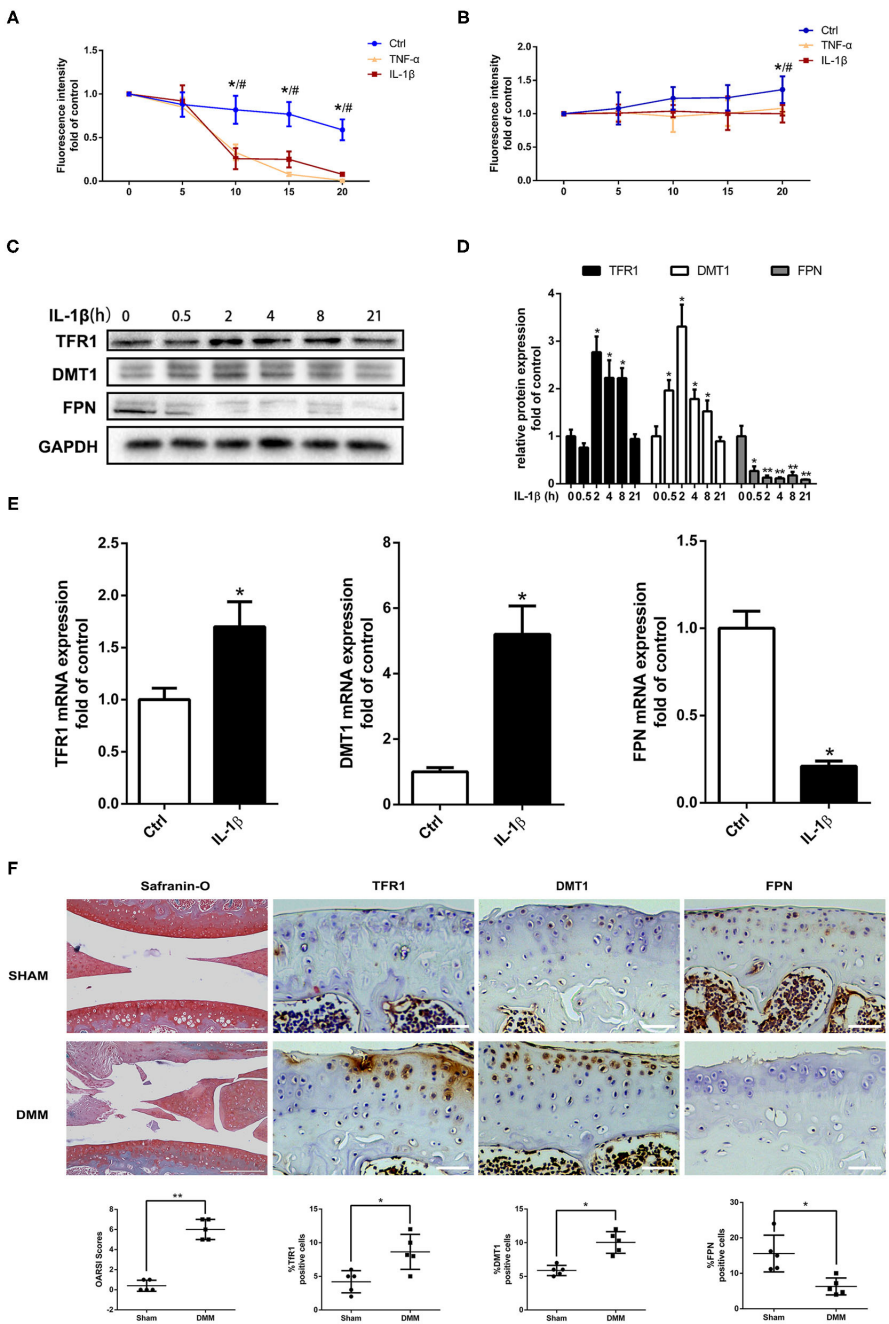
Pro-inflammatory cytokines IL-1β and TNF-α enhanced iron influx and attenuated iron efflux in chondrocytes via upregulating iron influx proteins, downregulating iron efflux protein. **(A)** Iron influx into chondrocytes was determined by the quenching and iron efflux **(B)** was determined by the reverse quenching of calcein fluorescence, which is an indicator of intracellular iron level. The fluorescence intensity represents the mean value of 25–30 cells from four separate fields at each time point (0–5–10–15–20 min) and is presented as the mean ± SD of six independent experiments. *P < 0.05 Ctrl vs. IL-1β group, ^#^*P* < 0.05 vs. TNF-α group. **(C)** Chondrocytes were treated with 10 ng/ml IL-1β for different time intervals and iron regulators, TFR1, DMT1, and FPN proteins expression were determined by western blot analysis. **(D)** The band density of TFR1, DMT1, and FPN were quantified and normalized to control. **(E)** Chondrocytes were treated with 10 ng/ml IL-1β for 24 h and RT-PCR was conducted to evaluated DMT1, TFR1, and FPN gene expression. The range of Ct was 18–30. All experiments were repeated three times independently. **(F)** Haematoxylin-Fast Green-Safranin-O staining and immunohistochemistry for TFR1, DMT1, FPN in the medial tibial plateau at 2 months after DMM surgery in mice of Ctrl group (*n* = 5 per group). The ratio of positive cells for TFR1, DMT1, and FPN were quantified under a microscope at 40X magnification using five sections from five mice. Scale bars=200 μM in Haematoxylin-Fast Green-Safranin-O images; Scale bars=50 μM in TFR1, DMT1, FPN staining images. Data are presented as mean ± SD. *P < 0.05; **P < 0.01. EZ=0.961 for OARSI in **(F)**; EZ=0.715 for TFR1 positive cells in **(F)**; EZ=0.856 for DMT1 positive cells in **(F)**; EZ=0.755 for FPN positive cells in **(F)**.

To elucidate the change of iron homeostasis and the associated iron regulators in OA, primary mouse chondrocytes were initially treated with 10 ng/ml IL-1β and TNF-α, fluorescence dye calcein was used to assess the iron uptake and outflow process in chondrocytes. As shown in Figure 4A, after perfusing with 0.1 mmol/L FAC, the fluorescence intensity significantly decreased in IL-1β or TNF-α treated chondrocytes compared with the control, indicating that IL-1β or TNF-α promoted chondrocytes iron uptake. For efflux investigation, the fluorescence intensity reverse was analyzed after 1 mmol/L DFO treatment, the fluorescence intensity was more weakly changed in IL-1β or TNF-α treated chondrocytes compared with control, indicating the iron efflux was inhibited (Figure 4B). There was no difference between IL-1β and TNF-α group in iron influx and efflux process.

To determine the mechanisms of iron transportation in IL-1β or TNF-α treated chondrocytes, western blot and RT-PCR analysis were performed to detect the iron transporters of TFR1, DMT1, and FPN. IL-1β treatment significantly promoted iron influx mediators, TFR1 and DMT1 expression and inhibited iron efflux mediator FPN expression (Figures 4C–E). To further determine the iron regulator proteins expression induced by pro-inflammatory cytokines in iron overloaded condition, primary chondrocytes were treated with 10 ng/ml IL-1β with or without 100 μM FAC for different time intervals and western blot assay was conducted to investigate the expression of TFR1, DMT1, and FPN. The expression of TFR1 and DMT1 in IL-1β + FAC group exhibited similar trend compared to the IL-1β group. IL-1β significantly promoted TFR1 and DMT1 proteins expression both in IL-1β and IL-1β+FAC group. For FPN, IL-1β inhibited FPN expression while IL-1β and FAC co-treatment promoted FPN expression (Supplementary Figure 1). Similar results were observed in our *in vivo* experiments. As shown in Figure 4F, Safranin-O results demonstrated that OA model was successfully established. Immunohistochemistry assay showed that TFR1, DMT1 expression were upregulated with about two-fold 8 weeks after DMM surgery when compared to sham control group. While FPN expression in the DMM group was about half of that in the sham control. In conclusion, our results indicate that pro-inflammatory cytokines could disrupt iron homeostasis via promoting TFR1, DMT1 expression and inhibiting FPN expression.

### Iron Overload Promoted Chondrocytes Apoptosis and Upregulation of Matrix-Degrading Enzymes in Chondrocytes

**Figure 5:**
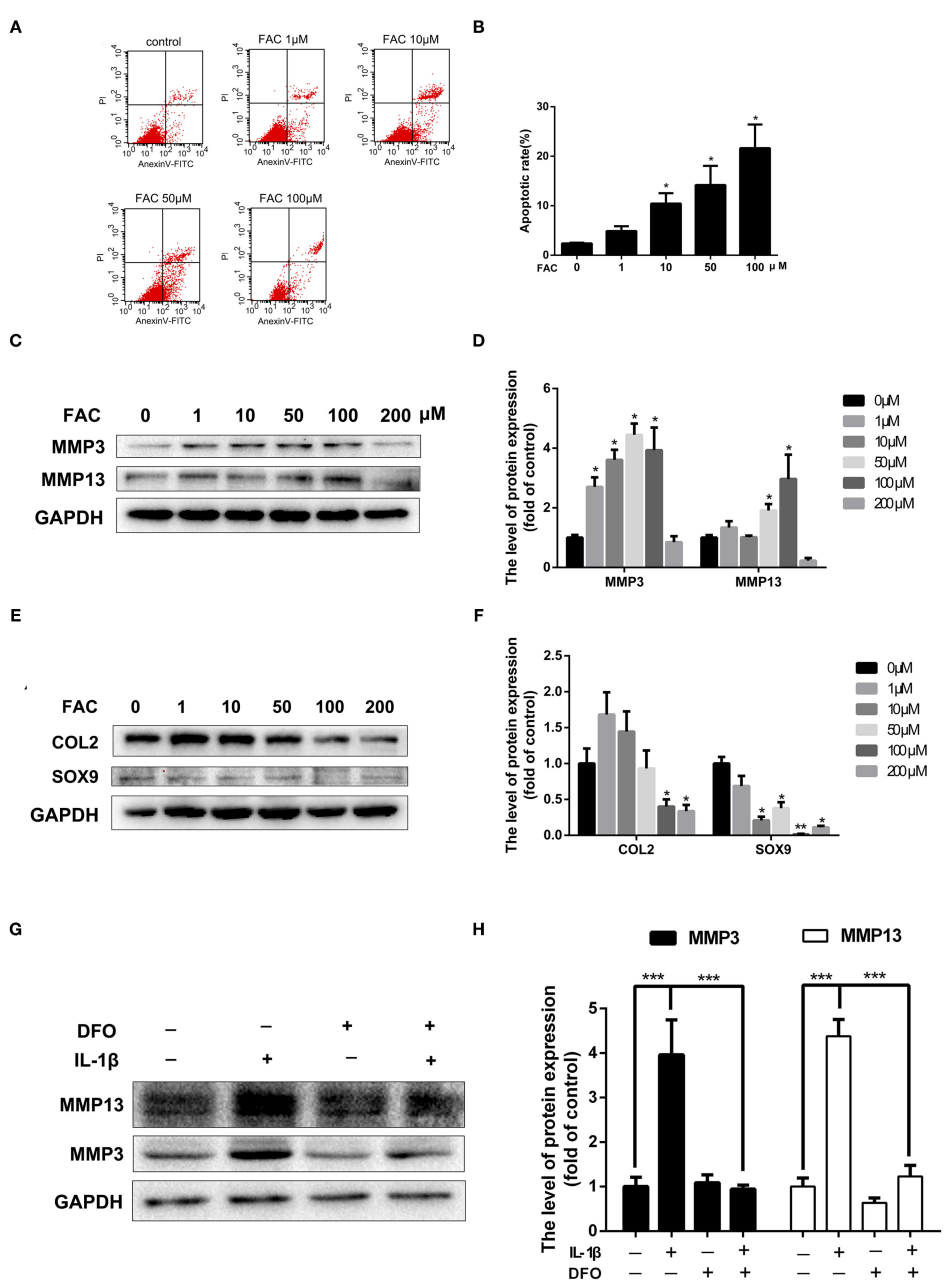
The detrimental effect of iron in OA pathogenesis. **(A,B)** Chondrocytes were treated with various concentrations of FAC and annexin V-FITC/PI flow cytometric analysis was conducted to evaluate apoptosis rate induced by FAC. **(C)** Chondrocytes were treated with various concentrations of FAC and MMP3, MMP13 proteins expression were determined by western blot analysis. **(D)** The band density of MMP3 and MMP13 were quantified and normalized to control. **(E)** Chondrocytes were treated with various concentrations of FAC and SOX9, COL2 proteins expression were determined by western blot analysis. **(F)** The band density of SOX9, COL2 were quantified and normalized to control. **(G,H)** Chondrocytes were treated with 10 ng/ml IL-1β with or without 100 μM DFO for 24 h and matrix degrading protein (MMP3, MMP13) expression were detected by western blot analysis. Data are presented as mean ± SD. All experiments were repeated three times independently. *P < 0.05; **P < 0.01; ***P < 0.001.

Elevated iron levels in chondrocytes stimulated by pro-inflammatory cytokines suggest their possible involvement in OA pathogenesis. To evaluate the detrimental effect of iron in chondrocytes, chondrocytes were treated with increasing concentrations of FAC for 24 h and Annexin V-FITC/PI double staining with flow cytometric analysis was performed. Chondrocytes apoptosis is closely linked to the OA development. As shown in Figures 5A,B, FAC promoted chondrocytes apoptosis in a dose dependent manner. Following treatment of increasing concentrations of FAC, the apoptotic cells were seen to raise from 2.3 to 21.6%. A hallmark of OA chondrocytes is their chondrocytes phenotype degeneration and increased production of matrix-degrading enzymes such as MMPs and ADAMTSs. As shown in Figures 5C,D, chondrocytes were treated with increasing concentrations of FAC for 24 h and western blot assay was conducted to examine MMP3 and MMP13 expression. Our results indicated that FAC promoted MMP3 and MMP13 expression in a dose dependent manner. FAC at concentration of 100 μM exhibited the most significant effect in promoting MMP3 and MMP13 expression. We then examined chondrogenic related proteins, such as SOX9 and COL2, following various concentrations of FAC treatment, we found that FAC significantly inhibited SOX9 and COL2 proteins expression (Figures 5E,F).

To further investigate the detrimental effect of iron in the production of MMPs in the presence of IL-1β, chondrocytes were treated with 10 ng/ml IL-1β for 24 h with or without 100 μM DFO. As shown in Figures 5G,H, obvious overproduction of MMP3 and MMP13 were observed after IL-1β treatment, while local iron depletion using DFO reversed IL-1β induced MMP3 and MMP13 upregulation. These results indicate that iron takes parts in chondrocytes apoptosis and cartilage ECM destruction in OA pathogenesis.

### Inhibition of DMT1 Restored IL-1β Induced Chondrocytes Cartilage Degeneration

**Figure 6:**
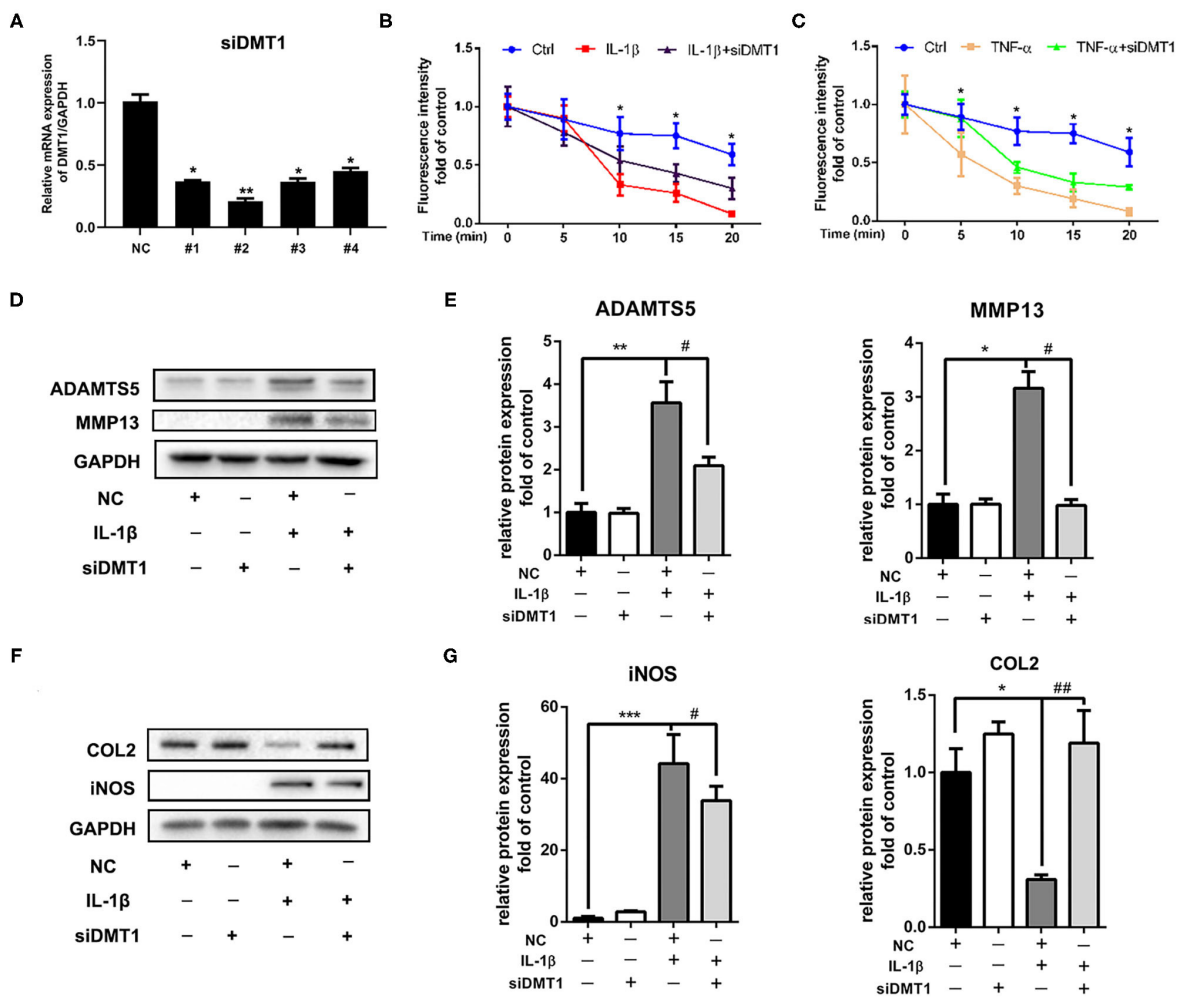
Inhibition of DMT1 restored IL-1β induced chondrocytes cartilage degeneration. **(A)** The effect of *Dmt1* silence was evaluated using RT-PCR analysis. **(B,C)** Chondrocytes were transfected with scrambled siRNA control or *Dmt1* siRNA, then treated with IL-1β or TNF-α for 24 h and iron influx into chondrocytes was determined by the quenching of calcein fluorescence. Data are presented as mean ± SD. *P < 0.05 IL-1β or TNF-α vs. *siDmt1* group. **(D–G)** Chondrocytes were transfected with *Dmt1* siRNA, then treated with IL-1β for 24 h. Protein levels of ADAMTS5, MMP13, COL2, and iNOS were determined by western blot **(D,F)** and quantification analysis **(E,G)**. Data are presented as mean ± SD. All experiments were repeated three times independently. *P < 0.05; **P < 0.01; ***P < 0.001 vs. scrambled siRNA control (NC); ^#^*P* < 0.05; ^##^*P* < 0.01 vs. IL-1β treatment group.

Cellular iron homeostasis is achieved mainly by controlling iron ions influx. We next investigated whether suppressing iron influx via inhibiting TFR1 or DMT1 expression could inhibit IL-1β induced cartilage degeneration. Interestingly, chondrocytes transfected with *Tfr1* siRNA failed to suppress IL-1β induced iNOS and MMP3 expression (Data are not shown). Chondrocytes were then transfected with *Dmt1* siRNA before IL-1β or TNF-α treatment. As shown in Figure 6A, *Dmt1* siRNA was synthesized and transfected into chondrocytes. Our results showed that inhibition of DMT1 significantly suppressed IL-1β or TNF-α induced iron influx (Figures 6B,C). As shown in Figures 6D–G, IL-1β caused notably increased protein levels of ADAMTS5, MMP13, iNos, and decreased expression of Collagen II, while inhibition of DMT1 markedly reversed these changes. Different tissue cells have different intracellular iron metabolism regulation modes and we hypothesize that DMT1 might be the main iron transporter in chondrocytes.

### Inhibition of DMT1 Suppressed IL-1β Induced MAPK and PI3K/AKT/NF-κB Pathway Activation

**Figure 7:**
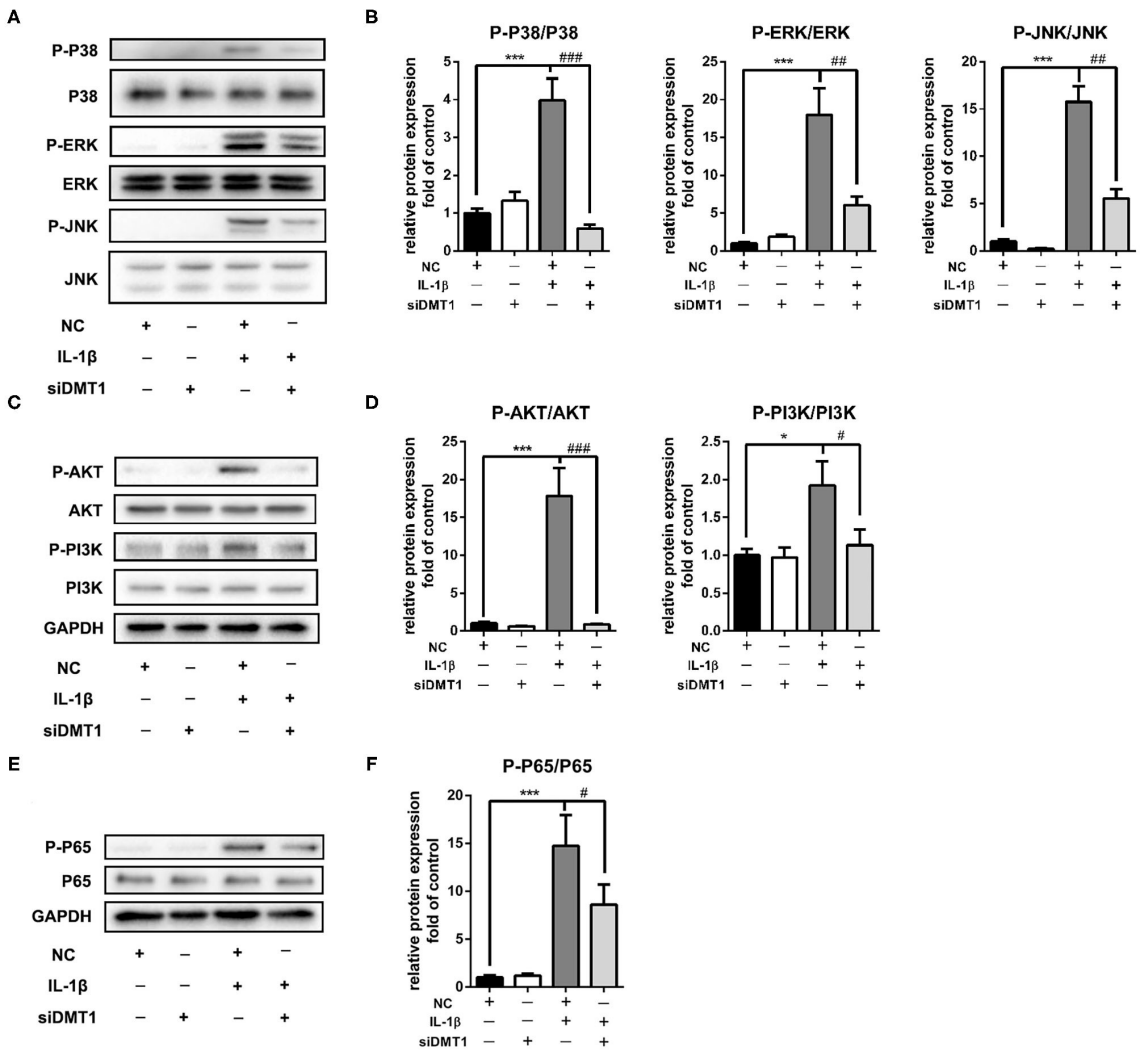
Inhibition of DMT1 suppressed IL-1β induced MAPK and PI3K/AKT/NFκB pathway activation. Chondrocytes were transfected with DMT1 siRNA, then treated with IL-1β for 30 min. The protein levels of P-P38, P38, ERK, P-ERK, JNK, P-JNK,P-AKT, AKT, P-PI3K, PI3K were determined by western blot **(A,C)** and quantification analysis **(B,D)**. **(E,F)** Chondrocytes were transfected with DMT1 siRNA, then treated with IL-1β for 15 min. The protein levels of P-P65, P65 were determined by western blot and the band density were quantified and normalized to control. Data are presented as mean ± SD. All experiments were repeated three times independently. *P < 0.05; ***P < 0.001 vs. NC; ^#^*P* < 0.05; ^##^*P* < 0.01; ^###^*P* < 0.001 vs. IL-1β treatment group.

Activation of the MAPK and PI3K/AKT/NF-κB signaling pathways is reported playing pivotal roles in OA development. To further understand the molecular mechanisms of the protective effects of DMT1 inhibition, we tested the two molecular pathways involved in OA after treatment of chondrocytes with *Dmt1* siRNA and IL-1β. MAPK is a common studied signaling pathway which is indicated playing important roles in OA development. As shown in Figures 7A,B, in accordance with previous published results, the MAPK pathway was activated after IL-1β treatment, while *Dmt1* siRNA blocked IL-1β induced phosphorylation of ERK, JNK and p38. PI3K/AKT/NF-κB pathway is a IL-1β-related signaling pathway, regulating cellular events including proliferation, hypertrophy, and apoptosis. As shown in Figures 7C–F, DMT1 inhibition via siRNA suppressed the phosphorylation of PI3K, AKT, P65 and thus inhibited the PI3K/AKT/NF-κB pathway activation, indicating DMT1 silence might protect against IL-1β induced ECM degradation via PI3K/AKT/NF-κB pathway.

## Discussion

Recent clinical studies indicated the link between iron overload and the incidence and progression of OA (van Vulpen et al., 2015b). However, no evidence available to date clearly shows the role of iron in the OA pathogenesis, nevertheless, the mechanism of iron takes parts in the onset and progression of OA remains unknown. In the present study we demonstrated that iron overload was a risk factor for OA pathogenesis. Mice with iron overload showed an accelerated OA progression associated with a greater cartilage degeneration, greater subchondral bone remodeling and an increased expression of MMPs. Moreover, we found that pro-inflammatory cytokines mediated iron homeostasis dysregulation played pivotal roles in iron overload induced OA progression. Cutting iron influx via inhibiting DMT1 could suppress IL-1β induced cartilage degeneration.

The iron overloaded mice model used in this study was established by parenteral administration of iron according to our previous studies and mimics hereditary hemochromatosis (HH) or transfusional iron overload (Jing et al., 2019). As expected, iron overloaded mice showed obvious hemosiderin deposition in the subchondral bone marrow and synovial tissue which is similar to what is seen in human. Synovial iron accumulation was found in human HH patients and in patients with rheumatoid arthritis (van Vulpen et al., 2015a). Increased concentrations of iron were also reported in the synovial fluid of OA, although to a lesser extent than in that of RA patients. It is thought that increased iron arises from blood that enters the joint by microbleeding or oozing from an inflamed synovial membrane (Ogilvie-Harris and Fornaiser, 1980). This could explain the results in our study that DMM operated knees of iron overloaded mice showed greater hemosiderin deposition compared to control group. In the present study we found that the DMM operated knees of iron overloaded group exhibited accelerated cartilage degeneration and higher expression of ADAMTS5, MMP13 compared to control group. According to the data from iron overloaded sham operated left knees, we hypothesize that iron overload alone seems not to be enough to cause OA in this time period. Iron overload is a risk factor for OA progression, microbleeding or synovial fluid excreting by trauma or mechanical overload lead to iron accumulation in afflicted joints. Elevated iron concentration in synovial fluid or hemosiderin deposition in cartilage tissue contributes to OA progression.

OA is primarily characterized by cartilage destruction. However, OA is a whole-joint disease with many pathological changes, among which subchondral bone remodeling plays important roles in the onset and progression of OA (Donell, 2019). Recent studies indicate that bone resorption is increased and subchondral bone thickness is decreased in the early stage of OA (Xie et al., 2019). The biomechanical changes in subchondral bone architecture could in turn result in subchondral bone collapse and sclerosis in the late-stage of OA (Stewart and Kawcak, 2018). In the present study, increased bone volume and trabecular thickness were observed in DMM operated knees of iron overloaded mice. Our results also showed increased osteoclasts in the iron overloaded mice. We hypothesize that iron overload induced osteoclast formation promote subchondral bone resorption and thus contribute to OA progression.

Our results indicated that iron overload played detrimental effects in OA progression, however, how excess iron damages chondrocytes under OA pathogenic conditions remains unknown. Excess free iron could act as a catalyst to produce large amount of ROS and lead to accumulation of lipid peroxides and ferroptosis (Kuang et al., 2020). Ferroptosis is a newly identified iron dependent cell death and we speculate that ferroptosis is involved in iron overload induced chondrocytes apoptosis. Iron homeostasis is delicately regulated in human body, but many risk factors, including age, mechanical load, inflammation could disturb iron homeostasis, and cause iron accumulation in tissues (Silva and Faustino, 2015). Pro-inflammatory cytokines, such as interleukin-1β (IL-1β) and tumor necrosis factor-a (TNF-a), are major components of OA inflammation which play key roles in OA cartilage damage (Wang and He, 2018). However, currently, controversial opinions exist about the involvement of pro-inflammatory cytokines in OA (van Dalen et al., 2017). In the present study, we found that IL-1β or TNF-α increased iron influx and decreased iron efflux, thus resulting in iron overload in chondrocytes. We also found that the expression of TfR1, DMT1 increased and FPN decreased, indicating that iron homeostasis and iron transporters take parts in OA pathogenesis. Iron overload is common in tissues of elderly people and has been implicated to be responsible for many diseases or pathological conditions. Different tissue cells have different intracellular iron metabolism regulation modes. In the present study, we found that DMT1 plays pivotal roles in pro-inflammatory cytokines induced chondrocytes iron overload and cartilage degeneration. Inhibition of *Dmt1* by siRNA not only inhibited IL-1β induced ADAMTS5, MMP13 and iNOS upregulation, but also restored IL-1β induced type II collagen inhibition.

The MAPK and PI3K/AKT/NF-κB signaling pathway were demonstrated to play vital roles in regulating inflammatory cytokines and ECM alterations in OA pathogenesis. IL-1β could activate all three MAPK pathways (ERK, P38, and JNK) as well as PI3K/AKT/NF-κB signaling cascade, subsequently promote the expression of catabolic factors, and contribute cartilage degeneration. Our results found that inhibition of DMT1 suppressed the phosphorylation of ERK, P38, JNK, PI3K, AKT, and P65. These results further demonstrated that DMT1 mediated iron influx plays pivotal roles in OA pathogenesis and DMT1 could be an attractive new target for OA treatment.

In conclusion, our results demonstrate that iron overload is a risk factor for OA progression. DMT1 mediated iron influx play important roles in chondrocytes iron homeostasis. Iron overload could promote chondrocytes apoptosis and the crucial effector matrix-degrading enzymes expression, thereby take parts in OA cartilage degeneration. Our results support the notion that local depletion of iron or cutting iron influx via DMT1 inhibition would be effective therapeutic approaches for the treatment of OA.

## Data Availability Statement

The raw data supporting the conclusions of this article will be made available by the authors, without undue reservation.

## Ethics Statement

The animal study was reviewed and approved by The Institutional Animal Care and Use Committee (IACUC) of Tongji Hospital.

## Author Contributions

XJ, XC, and KS conceived and designed the experiments. XJ, TL, JL, and XC performed the *in vitro* experiments. GW, KS, and XL performed the *in vivo* experiments. XJ, TD, ZJ, and JL analyzed the data. XJ and TD wrote the paper. All authors contributed to the article and approved the submitted version.

## Conflict of Interest

The authors declare that the research was conducted in the absence of any commercial or financial relationships that could be construed as a potential conflict of interest.
