# Supplementary figures and images for: Iron Overload Is Associated With Accelerated Progression of Osteoarthritis: The Role of DMT1 Mediated Iron Homeostasis
Source: Front Cell Dev Biol. 2021 Jan 5;8:594509. doi: 10.3389/fcell.2020.594509 (PMC7813682; doi:10.3389/fcell.2020.594509)

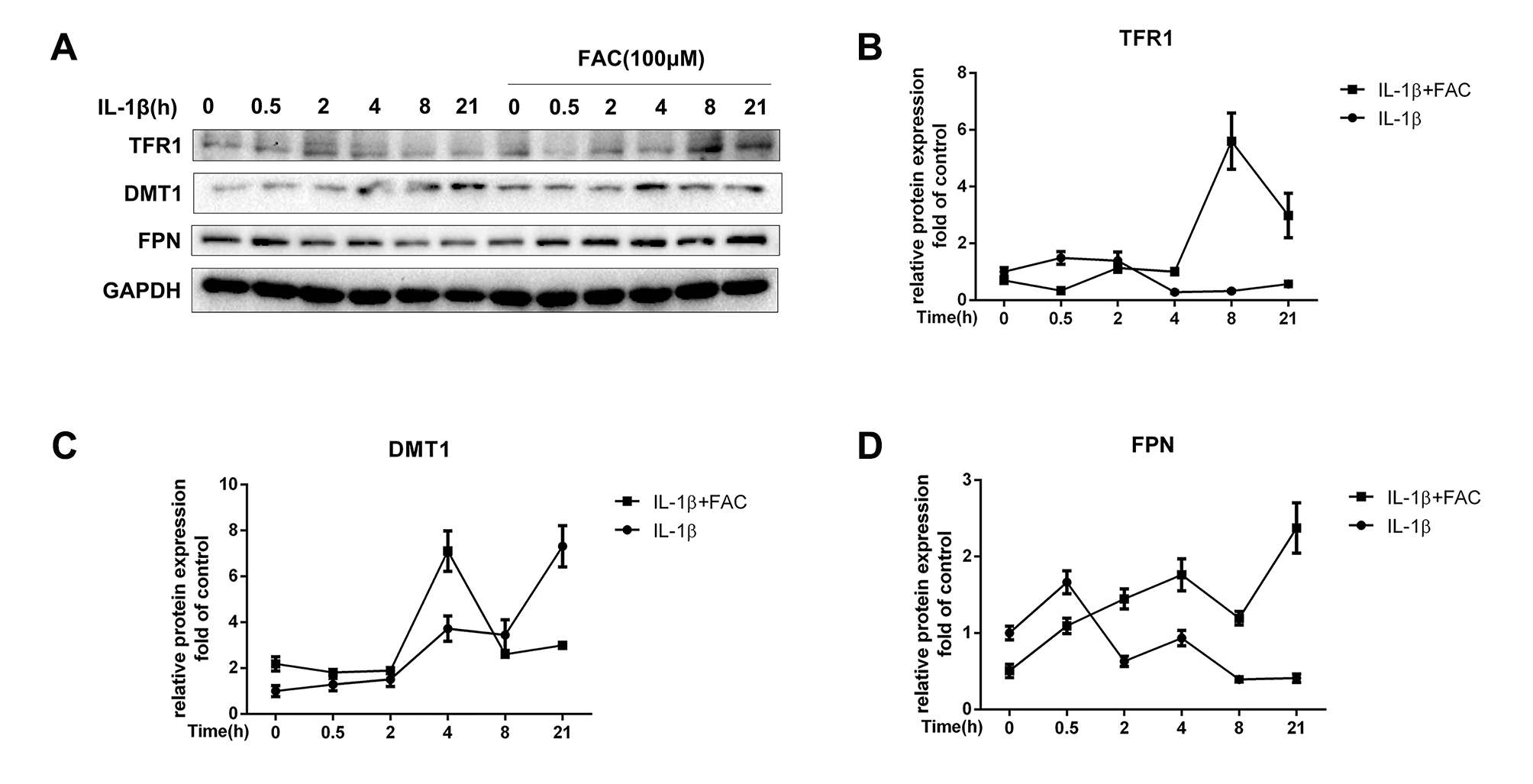

Supplement: Supplementary file 1 [file Image_1.TIF]
